# Supplementary material for: Urinary Metabolomics Study on the Protective Role of Cocoa in Zucker Diabetic Rats via 1H-NMR-Based Approach
Source: Nutrients. 2022 Oct 4;14(19):4127. doi: 10.3390/nu14194127 (PMC9572671; doi:10.3390/nu14194127)
Supplement: Supplementary file 1 [file nutrients-14-04127-s001.zip › nutrients-1945542-supplementary.pdf]

Supporting information:

Urinary metabolomics study on the protective role of cocoa in Zucker diabetic rats via ^{1}H-NMR-based approach

Elisa Fernández-Millán[1,2], Sonia Ramos[3], David Álvarez-Cilleros[3], Sara Samino[2,4], Nuria Amigó[2,4,5], Xavier Correig[2,4,6], Monica Chagoyen[7], Carmen Álvarez[1,2], María Ángeles Martín[2,3].

Supplementary table S1. Composition of the experimental control and cocoa-rich diets.

Component (g/Kg dry weight)	Control	Cocoa
Casein	140	140
Dextrose	155	155
Sucrose	100	100
Fat	40	40
t-BHQ (*tert*-butylhydroquinone)	0.008	0.008
Mineral mix.	35	35
Vitamin mix.	10	10
L-Cys	1.8	1.8
Cholin bitartrate	2.5	2.5
Cellulose	100	66
Starch	415.7	349.7
Cocoa powder	-	100
Energy (KJ/Kg diet)	15048	15048

Supplementary table S2. Metabolites identified in the urine of lean and diabetic rats.

L-Alanine	HMDB0000161
Dimethylamine	HMDB0000087
Formic acid	HMDB0000142
D-Glucose	HMDB0000122
1-Methylnicotinamide	HMDB0000699
Azelaic acid	HMDB0000784
Suberic acid	HMDB0000893
Pimelic acid	HMDB0000857
Urea	HMDB0000294
Tartaric acid	HMDB0000956
Pyruvic acid	HMDB0000243
Choline	HMDB0000097
Acetic acid	HMDB0000042
Phosphorylcholine	HMDB0001565
Creatinine	HMDB0000562
Acetoacetic acid	HMDB0000060
Hydroxyphenyllactic acid	HMDB0000755
Oxoglutaric acid	HMDB0000208
Creatine	HMDB0000064
Indoxyl sulfate	HMDB0000682
D_Unknow 3	-
D_Unknow 4	-
Phenylacetylglycine	HMDB0000821
Sucrose	HMDB0000258
Nicotinamide N-oxide	HMDB0002730
L-Lactic acid	HMDB0000190
L-Threonine	HMDB0000167
L-Valine	HMDB0000883
L-Leucine	HMDB0000687
L-Isoleucine	HMDB0000172
Hippuric acid	HMDB0000714
Ketoleucine	HMDB0000695

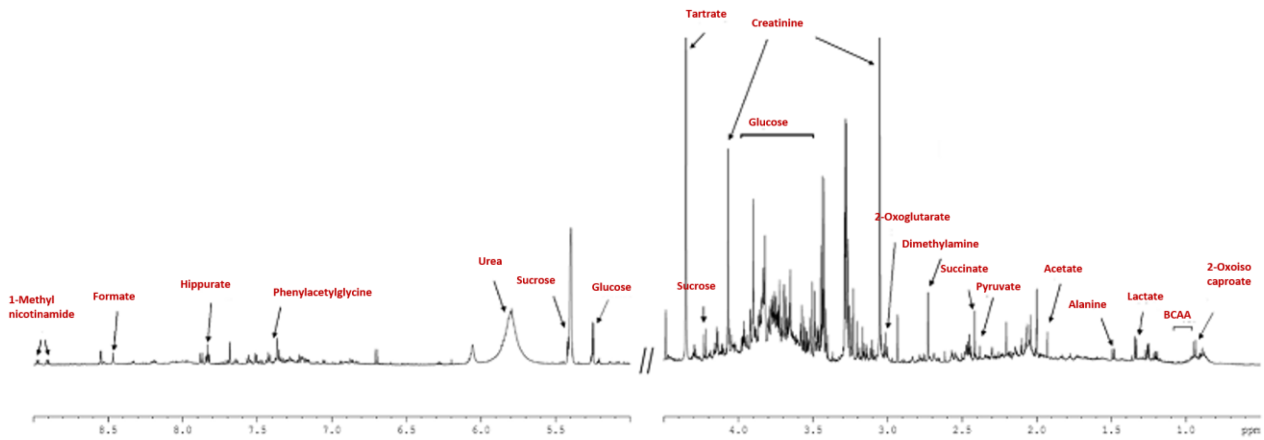

Figure S1. Representative 600-MHz ^{1}H-NMR spectrum of a urine sample including metabolite assignment.
